# Neutral lipid alterations in Human Herpesvirus 8-infected HUVEC cells and their possible involvement in neo-angiogenesis

**DOI:** 10.1186/s12866-015-0415-7

**Published:** 2015-03-28

**Authors:** Fabrizio Angius, Sabrina Uda, Enrica Piras, Stefano Spolitu, Angela Ingianni, Barbara Batetta, Raffaello Pompei

**Affiliations:** Department of Biomedical Sciences, University of Cagliari, via Porcell 4, Cagliari, 09124 Italy

**Keywords:** Herpesvirus 8, Endothelial cells, Viral infection, Cholesteryl esters, Triglycerides, Lipid droplets, Angiogenesis

## Abstract

**Background:**

Human Herpesvirus 8 (HHV8), the causative agent of Kaposi’s sarcoma, induces an intense modification of lipid metabolism and enhances the angiogenic process in endothelial cells. In the present study, neutral lipid (NL) metabolism and angiogenesis were investigated in HHV8-infected HUVEC cells. The viral replication phases were verified by rtPCR and also by K8.1 and LANA immunostaining.

**Results:**

Lipid droplets (Nile Red) were higher in all phases and NL staining (LipidTOX) combined with viral-antigen detection (immunofluorescence) demonstrated a NL content increase in infected cells. In particular, triglyceride synthesis increases in the lytic phase, whereas cholesteryl ester synthesis rises in the latent one. Moreover, the inhibition of cholesterol esterification reduces neo-tubule formation mainly in latently infected cells.

**Conclusions:**

We suggest that a reprogramming of cholesteryl ester metabolism is involved in regulating neo-angiogenesis in HHV8-infected cells and plays a likely role in the high metastatic potential of derived-tumours.

## Background

The Human Herpesvirus 8 (HHV8) is well known for its peculiar tropism for endothelial cells and B lymphocytes [1-5]. In fact, it is the causative agent of Kaposi’s sarcoma (KS) and of several lympho-proliferative diseases, namely primary effusion lymphoma (PEL) and multicentric Castleman’s disease. KS lesions are characterized by neo-angiogenesis and the production of typical spindle cells of endothelial origin [6,7]. HHV8-infection of cultured endothelial cells induces profound changes in the cellular phenotype, which resembles the spindle shape of KS lesion cells [1]. Moreover, the virus induces relevant modifications in the behaviour of both primary and immortalized endothelial cells and causes an intense transcriptional reprogramming in human umbilical vein endothelial cells (HUVEC) [8]. It also stimulates the Warburg effect in latently infected TIME endothelial cells with an increase of glycolysis and glucose consumption [9] and an activation of hypoxia-induced factors [10]. HHV8 is believed to establish persistent infection for the duration of the host’s lifespan, with occasional switching to the lytic phase of infection [11,12]. In addition, it has been demonstrated that the HHV8 latency-associated nuclear antigen (LANA) prolongs the lifespan of primary HUVEC and enhances cell survival in both serum-deprived conditions and in the presence of apoptotic inducers [13,14]. There are numerous examples in the literature that describe the biochemical and metabolic alterations in infected cells [2,3,10,15]. It has recently been found that PEL and other non-viral lymphoma cells synthesize fatty acids from glucose at a higher rate and contain more lipid droplets (LDs) as compared to primary B cells. To sustain the requirements of the proliferating lymphoma cells, the newly synthesized fatty acids are rapidly stored in triglycerides (TGs) and/or incorporated into membrane lipids [16]. Other authors have demonstrated that the increase of lipid synthesis and accumulation in LDs is dependent upon HHV8-gene expression, even in the latent phase [17]. Cholesteryl esters (CEs) as well as TGs are a common component of LDs. They have been found in great numbers in several diseases, such as tumours, infections [18] and atherosclerosis. However, CE synthesis, their content, and possible role during HHV8-infection have never been investigated [19-21]. For this reason, this study focuses on both CE and TG synthesis and their content during both lytic and latent HHV8-infection phases. We extended our investigation to the late latent phase because HHV8-latency leads to Kaposi’s sarcoma and to other lymphoproliferative malignancies in humans, characterized by higher CE content. Furthermore, we also investigated whether the inhibition of CE synthesis could affect neo-angiogenesis, which represents the main biological property acquired by infected cells, and is responsible for the remarkable metastatic potential of Kaposi’s sarcoma.

## Methods

### Cells and viruses

A pool stock of HUVEC cells (Invitrogen, Life Technologies, UK) was grown in a M200 medium (Gibco, Life Technologies, UK) with low serum growth supplement (LSGS, Invitrogen, Life Technologies, UK). HUVEC cells were always kept in a semi-confluent state and were sub-cultured at least once a week. Prior to the experiments, the cells had been sub-cultured no more than 3 to 5 times. HHV8 permanently infected BC3 cells were kindly donated by Dr. Caselli [6] and were grown in a RPMI-1640 medium supplemented with 10% fetal calf serum (FCS) (Invitrogen, Life Technologies, UK). BC3 cells were used to produce 100x concentrated stocks of HHV8, as previously described [22,23]. The virus pellet was suspended in RPMI, filtered through a 0.22 μm filter and kept at -80°C until use. The quantitative analysis of virus genomes present in the stock preparation was obtained by a real-time polymerase chain reaction (qPCR). The purified cell-free inoculums contained an average number of 4.7 × 10^5^ copies of viral DNA/ml (data not shown). For cell infection, about 5.0 × 10^4^/ml HUVEC cells were seeded in 12 multi-well plates. The cells were infected with HHV8, concentrated at a multiplicity of at least 10-20 genomes per cell in a M200 medium containing 2 μg/ml of polybrene for 2 h at 37°C. After 24-48 h the infected cells were observed with a light microscope to detect the typical spindle cell morphology. Only cell monolayers with at least 70-80% of HHV8-infected cells were used for the experiments.

### RT-PCR

The presence of HHV8 transcription in HUVEC cells was analyzed by RT-PCR for the amplification of the orf26, orf50 and orf73 (LANA) genes. Total RNA was extracted with a pureLink RNA Mini kit (Life Technologies, UK) and treated with TURBO DNase (Applied Biosystems, UK) before the synthesis of cDNA with a Super Script VILO kit (Invitrogen, Life Technologies, UK). RT-PCR amplification was performed using 200 ng of total RNA extracted from the infected cells as previously indicated [23-25]. Primers and conditions for RT-PCR amplification were as follows: orf26 took place with primers orf26 fw 5′-GCCGAAAGGATTCCACCATTGTGCT-3′ and orf26 rev 5′- GGGCCCCGGCCGATATTTTGG-3′ for 40 cycles (15'' at 95°C, 1' at 60°C and 15'' at 72°C) plus 10' at 72°C of extension; orf50 amplification took place with primers orf50 fw 5′-CATGCAGCGGGGTGAGCCTG-3′ and orf50 rev 5′- AGCAGCCCGGCGGTATCGTA-3′ for 40 cycles (15'' at 95°C, 1′ at 60°C and 15′ at 72°C); orf73 amplification took place with primers orf73 fw 5′- ATCCTCGGGAAATCTGGTCT-3′ and orf73 rev 5′-TTCAGCGTTTCAGTGTCTGC-3′ for 40 cycles (15'' at 95°C, 1′ at 60°C and 15′ at 72°C) plus 10′ at 72°C of extension. The amplification of the housekeeping ß-actin gene (actb) was used as a control with primers actb fw 5′-CACCATTGGCAATGAGCGGTTC-3′ and actb rev 5′- AGGTCTTTGCGGATGTCCACGT-3′ for 40 cycles (15'' at 95°C, 1′ at 60°C and 15′ at 72°C). RT-PCR products were run in 2% agarose gels and visualized by ethidium bromide staining. Acquisition and image processing were performed by PhotoDoc-It Imaging System Digital (UVP, Cambridge, MA) and PhotoPaint expression (Corel, Ottawa, Canada).

### Western blot analysis

The cells were lysed at 4°C in a PBS buffer containing 10% SDS, 50 μg TRIS, 1 μM EDTA, pH 7.5, 50 μM DTT and a protease/phosphatase inhibitor cocktail, homogenized by a UP100H Compact Ultrasonic Laboratory Device (Hielscher Ultrasonic GmbH, Teltow, Germany). The protein content of each sample was determined by the BCA assay (Sigma-Aldrich, Milan, Italy) [26] and processed as previously described [27]. In particular, protein samples (12 μg/lane) were separated by electrophoresis (acrylamide precast gel; Bio-Rad, Laboratories Inc., Milan, Italy) and transferred to nitrocellulose, 0.45 μm pore size (Millipore, Milan, Italy) by standard electro-blotting procedure. The blots were pre-treated with a blocking solution, ChemiBLOCKER™ (Millipore, Milan, Italy) diluted 1:3 v/v with TBST (50 μM TRIS-HCl, pH 7.6, 0.15 M NaCl and 0.05% Tween-20) for at least 1 h at RT before the addition of the primary antibodies (dilution 1:200) for K8.1 A/B (mouse monoclonal), LN53 (rat monoclonal) and β-actin (goat polyclonal). After overnight incubation at 4°C, the primary antibodies were removed and appropriate horseradish peroxidase-conjugated secondary antibodies were added in a dilution range of 1:5000 for at least 1 h at RT. All the antibodies were purchased from SantaCruz Biotechnologies (Dallas, TX, USA). Proteins were detected by enhanced chemiluminescence (Millipore, Milan, Italy) and by exposure to X-ray film (Sigma-Aldrich, Milan, Italy) for various times. Densitometric quantification of the protein bands was then accomplished by Image J software (NIH Bethesda, MA, USA).

### Viral antigen immunodetection

The cells were processed for microscopy experiments 3, 14 and 24 days after viral infection. In order to carry out the experiments, both the controls and the HHV8-infected HUVEC cells were diluted from stock cultures, seeded (in triplicate) at a density of 2.0 × 10^5^ in 35 mm glass-bottomed dishes (MatTek, Ashland, MA, USA) and cultured at 37°C in a 5% CO_2_ incubator in a growth medium for 24 h. After fixation with methanol for 10 min, cells were processed for viral immunodetection using a validated immunofluorescence kit assay (Scimedx Corp., Denville, NJ, USA) as indicated by the manufacturer’s instructions. BC3 cells were used as the positive control.

### Immunocytofluorescence and neutral lipid staining

The cells were processed for microscopy experiments 3, 14 and 24 days after viral infection. Two series of experiments were carried out. Both the controls and HHV8-infected HUVEC cells were diluted from stock cultures and seeded at a density of 2.0 × 10^5^ in 35 mm glass-bottomed dishes (MatTek, Ashland, MA, USA) and cultured at 37°C in a 5% CO_2_ incubator in a growth medium. After fixation with 4% paraformaldehyde (PFA) for 10 min, some cell cultures were stained with 300 nM Nile Red (9-diethylamino-5H-benzo[*α*]phenoxazine-5-one; Fluka, Buchs, SG, Switzerland) in PBS [28-30] and observed, in order to visualize neutral lipids in situ. Nile Red is a fluorescent dye that differentially stains polar lipids (i.e. phospholipids) and neutral lipids such as CEs and TGs. Polar lipids display a red emission, while neutral lipids have a green emission. Red emission was observed with 540 ± 12.5 nm excitation and 590 LP nm emission filters. Green emission was observed with 460 ± 25 nm excitation and 535 ± 20 nm emission filters. Furthermore, the other series of cell cultures was used to perform composite staining by firstly using a new neutral lipid probe called HCS LipidTOX™ Red Neutral Lipid (Life Technologies Corporation, UK), followed by an immunocytofluorescence method for identifying K8.1 (mouse monoclonal) or LANA (rat monoclonal) antigens (Santa Cruz Biotechnology, Santa Cruz, CA) with FITC-conjugated secondary antibodies counterstained in the same cells with Hoechst 33258 (Sigma-Aldrich, Milan, Italy) for nuclei. HCS LipidTOX™ Red Neutral Lipid stain has an extremely high affinity for neutral lipid droplets and is best imaged with filter sets appropriate for Alexa Fluor® 594 dye or Texas Red® dye (ex577-em609 nm). The XY coordinates were annotated in order to recognize the same microscopic field. Treble staining allowed us to evaluate possible differences in neutral lipid content between the control and infected cells.

### Microscopy and imaging

Microscope observations were performed with an Olympus IX71 inverted wide-field fluorescence microscope (Olympus, Tokyo, Japan) fitted with a 20×/0.7 or 2.5×/0.075 plan apochromatic objective. Twelve bit-images were captured using a cooled CCD camera (PCO Sensicam, Kelheim, Germany), electronically coupled to a mechanical shutter interposed between the 100 W Hg lamp and the microscope so as to limit photo bleaching. In some cases the excitation light was attenuated with a 6% neutral density filter. Nominal image resolution was 0.3 and 2.4 μm/pixel for 20 and 2.5× objectives, respectively. Quantitative analysis of images was performed with the Image Pro Plus package (Media Cybernetics, Silver Springs, MD, USA). At least 10 microscopic fields and 200 cells were individually selected and measured for each experimental group. Calculations were made with Excel (Microsoft Co., Redmond, WA, USA). Normalized data represent the percentage of the mean density value (intensity per pixel) ± standard error (SE).

### Determination of CE and TG synthesis

HUVEC cells were incubated for 4 h in a medium containing [^14^C]-oleate bound to bovine serum albumin (BSA). To prepare the oleate-BSA complex, 3.7 MBq of [^14^C]-oleic acid in ethanol (specific activity 2.035 GBq/mmol) were mixed with 1.4 mg KOH, after which the ethanol was evaporated. PBS (1.5 ml) without Ca^2+^ and Mg^2+^, containing 4.24 mg BSA (fatty acid-free) was added and the mixture shaken vigorously. This solution was added to each well to a final concentration of 74KBq/ml. After incubation, the cells were washed with ice-cold PBS and lipids extracted with acetone. The cell lysate was prepared for measuring protein content (Lowry method). Neutral lipids were separated by thin layer chromatography (TLC), visualized by iodine vapors, and the incorporation of [^14^C]-oleate into CEs and TGs was measured in a liquid scintillation counter.

### Angiogenic activity assay

The capillary-like micro-tubule formation in both the control and HHV8-infected cells was performed as reported in the literature [6]. 100 μl of Geltrex matrix (Gibco, Life technologies, UK) were poured into the bottom of 24 well plates and left to solidify for 1 h at 37°C as indicated by the manufacturer. After this, 3.0 × 10^4^ cells were seeded in a complete M200 medium and incubated for 24 h in a CO_2_ incubator at 37°C. In some experiments the cells were incubated in a serum-free medium. The formation of capillary-like micro-tubules was checked under a light microscope after 6 and 24 h. The angiogenic index was calculated according to the literature [13]. In several experiments, the specific ACAT inhibitor, Sandoz 58035 (SZ; Sigma-Aldrich, Milan, Italy), was used at a concentration of 4 μM which, as previously reported for other cell types [18,27], was the dose that also totally inhibits cholesterol esterification in HUVEC cells (Table 1) without altering viability and cell growth. All the samples were prepared in triplicate and the experiments were repeated at least twice.

**Table 1:** **SZ inhibits the CE synthesis in HHV8-infected and control cells**

	**HUVEC**	**HHV8-HUVEC**	**BC3**
untreated	100.00 ± 19.51	100.00 ± 12.39	100.00 ± 13.65
SZ	*13.16 ± 8.90	*15.19 ± 7.22	*8.42 ± 3.77

Control and HHV8-infected HUVEC and BC3 cells (1.0x10^6^) were treated with Sandoz 58035 (SZ, 4 μM) for 24 h. Before the last 4 h, [^14^C]-oleate bound to BSA was added to the medium and cells were incubated for the remaining time. Subsequently, cells were washed with ice-cold PBS and lipids extracted with acetone. Neutral lipids were separated by thin layer chromatography (TLC), and the incorporation of [^14^C]-oleate into CEs was determined as described in Methods. Data were reported as percentage of DPM/10^6^ cells mean ± SE. Significance was set up when p < 0.05 (*) vs. respective control (t-test).

### Statistical analysis

Statistical analysis was performed with GraphPad Prism (GraphPad Software Inc. La Jolla, CA, USA) software and Statistica (StatSoft, Tulsa, OK, USA). All data were expressed as the mean ± SE of experiments in triplicate and analyzed by the t-student test or ANOVA, and LSD-Fisher as a post-hoc test when required. Data were considered significant when p < 0.05.

## Results

### Characterization of lytic and latent phases during long term HHV8 infection of HUVEC cells

**Figure 1:**
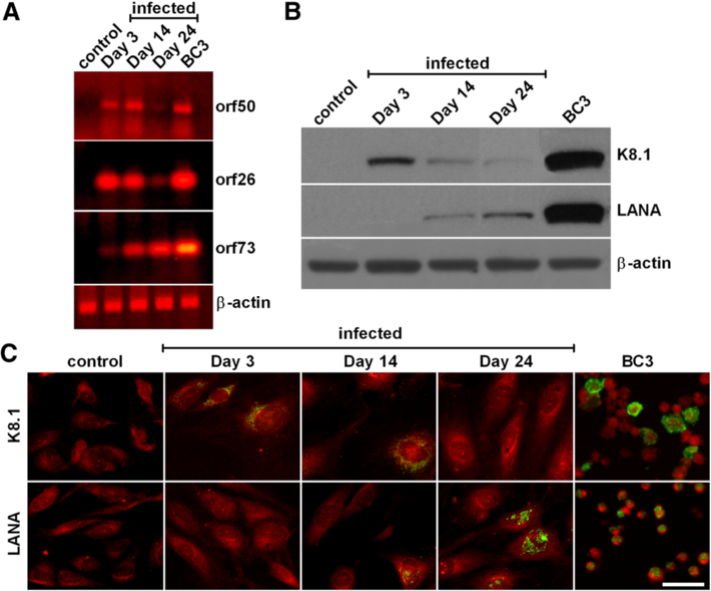
**Characterization of lytic and latent phases during long term HHV8 infection of HUVEC cells.** HUVEC cells were infected with HHV8, concentrated at a multiplicity of at least 10-20 genomes per cell in a M200 medium containing 2 μg/ml of polybrene for 2 h at 37°C. **Panel A**: On days 3, 14 and 24 post infection, 1.0 × 10^6^ cells were harvested and RNA-extracted for detection of lytic (orf26 and orf50) and latent (orf73) viral genes by RT-PCR. HHV8-infected BC3 cells were used as a positive control. The housekeeping β-actin gene was used as a control (for details see Methods). **Panel B**: At the indicated times (3, 14 and 24 days post infection) sub-confluent cells were harvested and processed as required for western blotting (for details see Methods). **Panel C**: 24 h before the indicated times (3, 14 and 24 days post infection), cells were seeded at a density of 2.0 × 10^5^ in 35 mm glass-bottomed dishes and cultured at 37°C in a 5% CO_2_ incubator in a growth medium. Thereafter, cells were washed and fixed for the immunofluorescence detection of lytic (K8.1) and latent (LANA) viral-antigens (green) as reported in Methods. The bar in the figure is 30 μm.

HHV8-infected cells were in good shape and continued to replicate for up to 6-8 weeks after infection, whereas control cells started to slow their growth and show signs of senescence after 4 weeks of culture. For this reason, our experiments were performed from 3 to 24 days after infection (Figure 1). On days 3 and 14, lytic genes *orf26* and *orf50* were both clearly expressed, whilst latent gene *orf73*, slightly expressed on day 14, was the only gene transcribed on day 24. Gene expression data were confirmed by western blotting analysis (Figure 1B) and immunofluorescence detection for lytic (K8.1) and latent (LANA) viral-antigens (Figure 1C). Indeed, on day 3 at least 70-80% of cells were K8.1-positive; on day 14 a mixed population of either K8.1- or LANA-positive cells was present, whereas on day 24 about 40-60% of cells were LANA-positive.

### Neutral lipid accumulation in lipid droplets in HHV8-infected HUVEC cells

**Figure 2:**
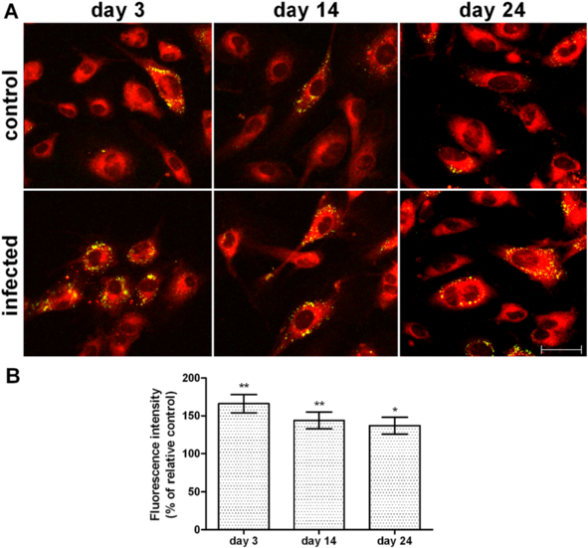
**Neutral lipid content in lipid droplets in HHV8-infected HUVEC cells.** HUVEC cells were infected with HHV8 as described in Figure 1. 24 h before the indicated times, cells were seeded at a density of 2.0 × 10^5^ in 35 mm glass-bottomed dishes and cultured at 37°C in a 5% CO_2_ incubator in a growth medium. On days 3, 14 and 24 post infection, cells were fixed and stained with Nile Red (for details see Methods), reported as green/yellow dots in the figure **(panel A)**. The bar in the figure is 30 μm. **Panel B** represents the quantitative analysis of Nile Red green fluorescence intensity. At least 200 cells were individually selected and analyzed for each experimental group. Normalized data represent the percentage of the mean density value (intensity per pixel) ± standard error (SE). Significance was set up when p < 0.05 (*) or p < 0.01(**) vs. respective control (ANOVA and Fischer’s LSD as post hoc test).

**Figure 3:**
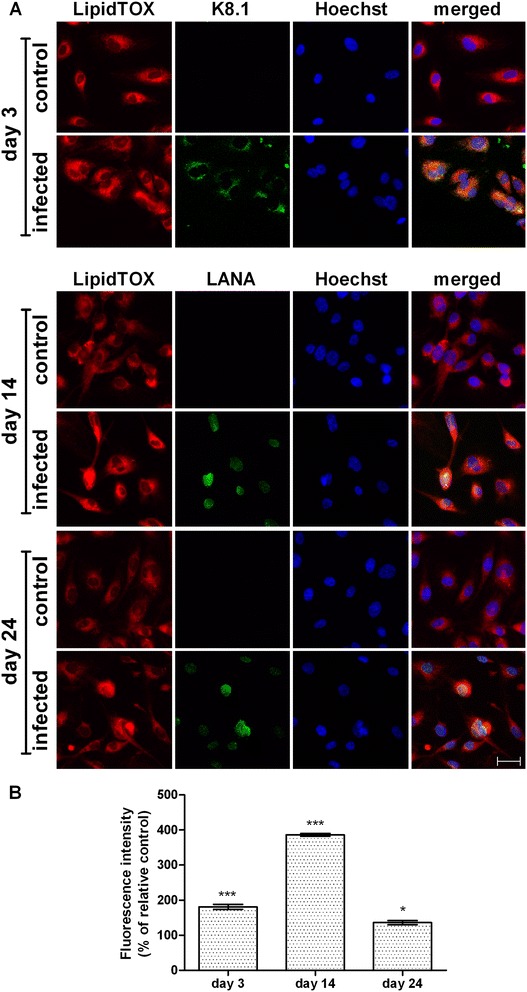
**Neutral lipid detection and quantification in HHV8-infected HUVEC cells by LipidTOX dye.** HUVEC cells were infected with HHV8 as described in Figure 1. 24 h before the indicated times, cells were seeded at a density of 2.0 × 10^5^ in 35 mm glass-bottomed dishes and cultured at 37°C in a 5% CO_2_ incubator in a growth medium. On days 3, 14 and 24 post infection, cells were fixed and treble-stained with firstly HCS LipidTOX™ Red Neutral Lipid stain (red), followed by an immunocytofluorescence method for identifying lytic (K8.1) and latent (LANA) viral-antigens with FITC-conjugated secondary antibodies (green) in the same cells counterstained with Hoechst 33258 for nuclei (blue), for details see Methods **(panel A)**. The bar in the figure is 30 μm. **Panel B** represents the quantitative analysis of HCS LipidTOX™ Red Neutral Lipid stain fluorescence intensity. This method allowed the quantification of the neutral lipids in LDs in HHV8-infected cells alone. At least 10 microscopic fields were individually analysed for each experimental group. Normalized data represent the percentage of the mean density value (intensity per pixel) ± SE. Significance was set up when p < 0.05 (*) or p < 0.001 (***) vs. respective control (ANOVA and Fischer’s LSD as post hoc test).

Figure 2A shows a remarkable increase of neutral lipids in LDs in all the HHV8-infected cells when compared to the respective control. As demonstrated by imaging analysis, the highest increase was observed on day 3 (Figure 2B). As is evident from the images, there is a heterogeneous distribution of LDs throughout the cells, probably due to the mixed population of infected/uninfected cells. In order to ascertain whether the neutral lipid increase was a peculiarity of the infected cells, we next used a double stain for neutral lipids (LipidTOX, red) combined with FITC-conjugated antibodies (green) for the detection of viral-antigens, namely K8.1 on day 3 and LANA on days 14 and 24 (Figure 3A). Imaging analysis of the merged images demonstrated that LDs were definitely higher in infected-cells (Figure 3B). In particular, when the LDs were only evaluated in infected cells, the strong increase of LDs was more evident on day 14 (p < 0.001). The differences between the infected and control cells were statistically significant (p < 0.05).

### Synthesis of TGs and CEs in HHV8 infected HUVEC cells

**Figure 4:**
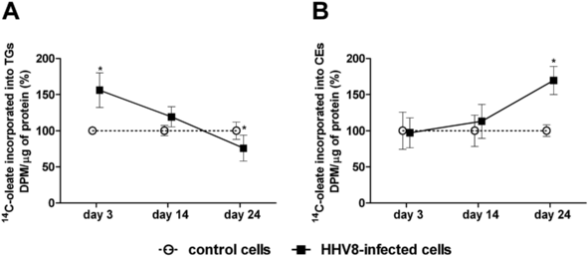
**TG and CE synthesis in HHV8-infected and control HUVEC cells.** HUVEC cells were infected with HHV8 as described in Figure 1. On days 3, 14 and 24 post infection, 1.0 × 10^6^ cells were incubated for 4 h in a medium containing [^14^C]-oleate bound to bovine serum albumin (BSA). Subsequently, cells were washed with ice-cold PBS and lipids extracted with acetone. Neutral lipids were separated by thin layer chromatography (TLC), and the incorporation of [^14^C]-oleate into TGs **(panel A)** and CEs **(panel B)** was determined as described in Methods. Data were reported as mean ± SE. Significance was set up when p < 0.05 (*) vs. respective control (t-test).

Neutral lipids stored in LDs are variably constituted by TGs and CEs. To evaluate their major component in infected cells, we measured TG and CE synthesis at the different phases of infection. As demonstrated in Figure 4A, on days 3 and 14, TG synthesis was higher than the respective control, but significant only on day 3 (p < 0.05), whereas on day 24, TG synthesis significantly decreased (p < 0.01). However, CE synthesis did not change on days 3 and 14, whereas, on day 24 when all the infected cells were in a latent state, CE synthesis was about 69% higher than the respective control (Figure 4B, p < 0.001).

### CE synthesis inhibition induces impairment of HUVEC cell neo-angiogenic activity

**Figure 5:**
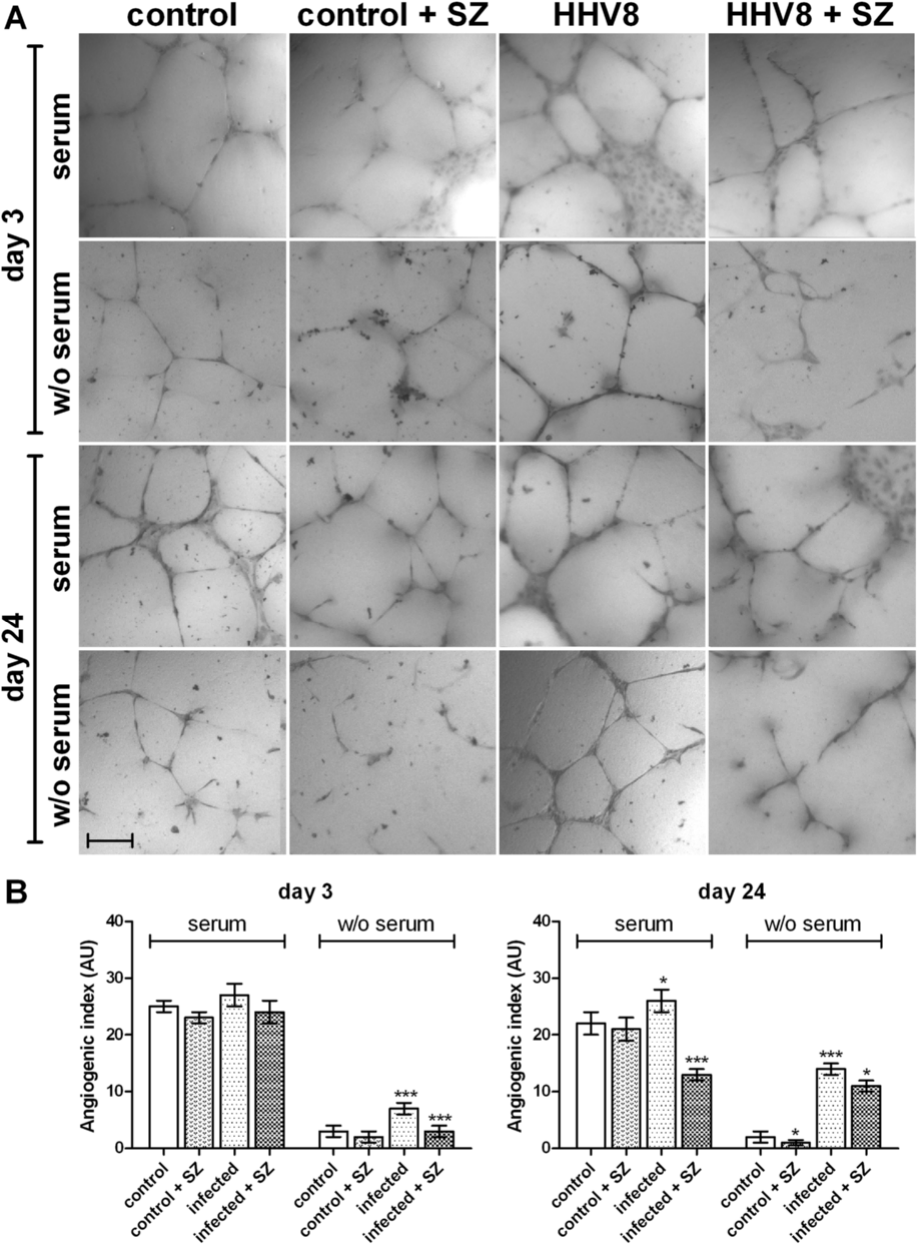
**Capillary-like vascular micro-tubule formation in HHV8-infected and control HUVEC cells.** HUVEC cells were infected with HHV8 as described in Figure 1. 24 h before the indicated times, 3.0 × 10^4^ cells were seeded in plates previously coated with Geltrex matrix and cultured in a complete or serum-free M200 medium in a CO_2_ incubator at 37°C. The specific ACAT inhibitor Sandoz 58035 (SZ, 4 μM) was added in some experimental groups. Capillary-like microtubule formation in the control and HHV8-infected HUVEC cells was performed as described in the Methods section. **Panel A** shows the light microscope images of micro-tubule formation in control, lytic and latent HHV8-infected HUVEC cells cultured in medium with or without (W/O) fetal calf serum (FCS). The bar in the figure is 240 μm. **Panel B** represents the angiogenic index quantification (for details, see Methods). All the samples were prepared in triplicate and the experiments were repeated at least twice. Data were reported as mean ± SE. Significance was set up when p < 0.05 (*) or p < 0.001 (***) vs. respective control (t-test).

In order to verify whether neutral lipids, specifically CEs, could somehow also be involved in the peculiar modifications induced by lytic or latent HHV8 infection, we evaluated their possible role in neo-angiogenesis, which is typically enhanced in HHV8-infected cells. In fact, the neo-angiogenic properties of HHV8 are necessary for the formation of the characteristic lesions of Kaposi’s angiosarcoma [1-6]. In the angiogenic activity assay, both control and lytic (day 3) or latent (day 24) HHV8-infected cells produced micro-tubules within 24 h (Figure 5A and B). The specific inhibitor of CE synthesis SZ 58035 significantly reduced tubule formation in infected cells on day 24 (p < 0.001) but not during the lytic infection (day 3). Interestingly, control cells grown in a serum-free M200 medium were not able to produce complete capillary micro-tubules, whilst HHV8-infected cells still formed regular and almost normal tubules (p < 0.05). Furthermore, in these conditions (Figure 5A and B) SZ was also able to dramatically reduce tubule formation (p < 0.001) in both lytic and latent infection, as confirmed by evaluation of the angiogenic index (Figure 5B) [13].

## Discussion

HHV8 is known for its endothelial tropism and is generally related to proliferative diseases, mainly KS. Recently, dysfunctional glucose metabolism in vitro [23], and a high prevalence of HHV8 infection in subjects with diabetes mellitus [24] have been reported. Neutral lipid increase in LDs, mainly TGs, has been reported in several herpes virus infections, and considered an effect of the reprogramming induced by the viral infection to consent virion production [31]. A few reports have described the presence of HHV8 in plaque lesions, but the possible correlation with atherosclerosis has yet to be clarified [32,33]. In the present study, we investigated neutral lipid metabolism and LD content during the lytic and latent phases of endothelial HHV8-infection. In fact, most studies have investigated the effect of cell reprogramming on lipids in the first days of infection. We present a model that allows the HHV8-infectious effect on cell metabolism to be investigated from the lytic to the late latent phase for up to more than three weeks, which is particularly suitable for HHV8-infection of endothelial cells. Even though, as shown by immunofluorescence, 40-60% of cells still appeared to be infected on day 24 after infection, it was not possible to prolong the experiment any further because the cells gradually lost their viral episome and rapidly started to show some signs of senescence (data not shown). TG synthesis was increased in the lytic phase. The fact that cholesterol esterification was not significantly modified for up to 14 days suggests that the higher LD content was due to TG content. Conversely, TG synthesis was lower, whereas cholesterol esterification underwent a remarkable increase in the latent phase, suggesting that, while TGs are prominent in the lytic phase, CEs are the prominent neutral lipid fraction in the late latent phase. It is worth noting that we performed our lipid synthesis analysis in a mixed population of infected/uninfected cells, as demonstrated by immunofluorescence. Based on this consideration, it is likely that our results could be underestimated. To overcome this problem, we double-stained neutral lipids (LipidTOX) and viral-antigens (immunofluorescence) in the same cells, and the results confirmed that neutral lipids increased only in infected cells. An increase of TG-enriched LDs during the lytic phase of viral infection has often been reported for HCV virus. In fact, it has been suggested that the virus uses TG-enriched LDs as a platform for the assembly of nascent virions, and that the HCV core protein is a main player in the manipulation of these organelles [34,35]. Overall, these observations highlight that a range of viruses (HBV, GBV-B, Dengue virus) have evolved mechanisms to interact with LDs and possibly to subvert the function of these organelles, so as to use them as a platform for viral particle assembly [31]. Accordingly, our results support the fact that TG synthesis and LD increase are mainly necessary during active virus replication. On the contrary, in the latent phase of HHV8-infection, LD content seems to be mainly constituted by CEs, as suggested by the high rate of cholesterol esterification. High CE content and cholesterol esterification increase are frequently reported in cancer cells and tumours [18,27]. The storage of CEs has been related to the large amount of cholesterol needed by malignant cells to sustain membrane biogenesis. Although the real significance of CEs in cancer cells is far from being clarified, CEs have recently been related to the aggressiveness and metastatic properties of prostate cancer [36,37]. For the first time, we observed the increase of cholesterol esterification in the latent phase, which can be considered equivalent to persistent infection in humans, and as leading to KS. The most common pathogenic effect expressed by HHV8 in KS is represented by the angiogenic activity of infected cells. Accordingly, angiogenesis is one of the major effects produced by HHV8-chronic infection in vitro and, just as in cancer cells, still present even in serum-deprived conditions [13]. For these reasons, we inhibited CE formation using a specific ACAT inhibitor (Sandoz 58035), which is able to inhibit cholesterol esterification up to 90%, but does not affect TG synthesis. In contrast with the control, HHV8-infected cells produced micro-tubules even in serum-deprived conditions. Interestingly, the inhibition of CE formation was accompanied by a remarkable reduction of micro-tubules in HHV8-infected cells.

## Conclusions

HHV8-infection induces an enhancement of neutral lipid synthesis and the accumulation of LDs in HUVEC cells. The TG increase in the lytic phase is probably related to peculiar metabolic viral requirements. On the other hand, CEs seem to be closely related to the angiogenic properties of the infected cells, mainly in the latent phase of infection, suggesting that these neutral lipids may, in some way, be functionally involved in regulating the malignant process. These findings suggest that the use of cholesterol esterification inhibitors on HHV8-derived tumours could be a useful therapeutic tool in KS. In fact, angiogenesis inhibition may help to prevent the occurrence of the diffusion of angiosarcoma and metastasis.
